# Relation of left atrial appendage closure devices to topographic neighboring structures using standardized imaging by cardiac computed tomography angiography

**DOI:** 10.1002/clc.23136

**Published:** 2018-12-21

**Authors:** Simon Lindner, Michael Behnes, Annika Wenke, Benjamin Sartorius, Wulf Dieker, Uzair Ansari, Muharrem Akin, Thomas Bertsch, Kambis Mashayekhi, Nils Vogler, Holger Haubenreisser, Stefan O. Schoenberg, Martin Borggrefe, Ibrahim Akin

**Affiliations:** ^1^ First Department of Medicine, University Medical Center Mannheim (UMM), Faculty of Medicine Mannheim University of Heidelberg, European Center for AngioScience (ECAS), and DZHK (German Center for Cardiovascular Research) Mannheim Germany; ^2^ Department of Cardiology and Angiology Hannover Medical School Hannover Germany; ^3^ Institute of Clinical Chemistry, Laboratory Medicine and Transfusion Medicine, General Hospital Nuremberg Paracelsus Medical University Nuremberg Germany; ^4^ Clinic for Cardiology and Angiology II University Center Freiburg Bad Krozingen, University of Freiburg Bad Krozingen Germany; ^5^ Institute of Clinical Radiology, University Medical Center Mannheim (UMM), Faculty of Medicine Mannheim University of Heidelberg Mannheim Germany

**Keywords:** cardiac computed tomography angiography, left atrial appendage closure, LOVE, neighboring structures, standardized imaging protocol

## Abstract

**Background:**

Although left atrial appendage (LAA) anatomy and topographic relations are well understood, little is known about the impairment of neighboring structures (NBS) by an implanted left atrial appendage closure (LAAC) device. This prospective longitudinal observational study for the first time describes distances of implanted LAA closure (LAAC) devices to NBS using a standardized imaging protocol of cardiac computed tomography angiography (cCTA).

**Hypothesis:**

cCTA imaging is an eligible tool for post‐implantation evaluation of LAAC devices and their relation to neighboring structures.

**Methods:**

cCTA data sets of consecutive patients 6 months after successful LAAC were acquired on a third generation dual‐source CT system and reconstructed with a slice thickness of 0.5 mm. The standardized multi‐planar reconstruction LAA occluder view for post‐implantation evaluation (LOVE) algorithm was used to measure the distances to NBS in relation to LAA morphology and implanted LAAC devices.

**Results:**

A total of 48 patients (median age 80 years, 25% female) were included. Left upper pulmonary vein and circumflex artery were generally closest to occlusion devices (median 2.9 and 2.8 mm, respectively). AMPLATZER AMULET devices were closer to the mitral valve annulus than WATCHMAN devices (6.6 mm (inter quartile range [IQR] 4.9‐8.6) vs 10.9 mm (IQR 7.4‐14.0), *P* = 0.001). Distances to the left upper pulmonary vein were affected by LAA morphology, with cauliflower type having the closest proximity (1.7 mm [IQR 1.0‐3.4], *P* = 0.048).

**Conclusion:**

A standardized cCTA imaging protocol is an eligible tool to accurately measure distances to NBS. Left upper pulmonary vein and circumflex artery are closest to LAAC devices and could thus be most prone to impairment.

## 1 INTRODUCTION

In non‐valvular atrial fibrillation (AF), the left atrial appendage (LAA) has been described as the main site of thrombus formation.^1^, ^2^ This provides the rationale for direct targeting of this site by interventional LAA closure (LAAC) to reduce the risk of ischemic stroke. Especially, as the effectiveness of this procedure has been demonstrated by follow‐up of the PROTECT‐AF study compared to conventional anticoagulation therapy,^3^ LAAC is becoming an established treatment alternative for stroke prevention in AF patients.

LAA anatomy and topographic relations to neighboring structures (NBS) are well described, thanks to post‐mortem dissection and increasing availability of high‐resolution cross‐sectional imaging. In contrast, understanding of the effects of device implantation on those NBS is more hypothetical than evidence‐based. Because of high variability in LAA morphology and anatomical correlations to NBS, prevention of device‐derived complications confronts clinicians with problems that differ in each case. Described as possible structures especially at risk because of their proximity to the LAA have been the mitral valve annulus (MVA), left pulmonary artery (LPA), left upper pulmonary vein (LUPV) and circumflex artery (Cx).^4^, ^5^, ^6^, ^7^ Furthermore, case reports of occlusion of Cx,^8^ perforation of LPA^9^, ^10^ and pulmonary vein compression^11^ have been published. We therefore see a need for further assessment of the relations of implanted LAAC devices to NBS.

Assessment of NBS can be performed through transesophageal echocardiogram (TEE).^12^ However, cardiac computed tomography angiography (cCTA) may reveal the potential as an even more accurate imaging modality,^7^, ^13^ as it offers higher resolution images for measuring distance to and impairment of LAA NBS. Recently, a standardized cCTA imaging protocol has been proposed by our working group,^13^, ^14^ which could prove useful in the post‐implantation evaluation of implanted LAAC devices. Higher resolution and non‐invasiveness of cCTA evaluation of LAAC compared with TEE, paired with higher reliability and objectivity of a standardized protocol are arguments in favor of cCTA.^13^, ^14^

Therefore, the present study evaluates the distances of NBS to the implanted device using the standardized cCTA protocol—LAA occluder view for post‐implantation evaluation (LOVE) ‐ 6 months after successful LAAC. Distances to NBS are assessed in relation to types of occlusion devices as well as the LAA morphologies.

## 2 MATERIALS AND METHODS

### 2.1 Study population

This is a prospective, non‐randomized, observational, longitudinal single‐center study. Consecutive patients with non‐valvular AF and indication for oral anticoagulation due to a CHA_2_DS_2_‐VAsc score ≥ 2 undergoing LAAC device implantation between June 2014 and December 2017 were included. Accordant to the 2016 guidelines of the European Society of Cardiology (ESC),^15^ sex category as independent risk factor was excluded from the original CHA_2_DS_2_VASc score. Inclusion criteria were a relative or absolute contraindication for oral anticoagulation, which was major or recurrent bleeding, HAS‐BLED score ≥ 3 or intolerance to oral anticoagulation and age ≥ 18. Exclusion criteria were a treatable cause or a single episode of AF, planned catheter ablation of AF or electrical cardioversion within 30 days prior or after LAAC, myocardial infarction within the last 3 months, congestive heart failure of New York Heart Association (NYHA) stage IV, atrial septum defect (ASD) or interventional or surgical occlusion of ASD, mechanical heart valve, status after heart transplant, intracerebral bleeding within the last 3 months, symptomatic carotid stenosis, transient ischemic attack (TIA) or stroke within the last 30 days, acute infection, existing or planned pregnancy, and existing cardiac thrombus.

LAAC device implantation was performed by experienced interventional cardiologists. The selection of one of the two devices—WATCHMAN (Boston Scientific, Natick, Massachusetts) or AMPLATZER AMULET Cardiac Plug (St. Jude Medical, St Paul, Minnesota)—was based on individual anatomic considerations. For smaller and flat left atrial appendages AMPLATZER AMULET devices were primarily considered. Details of the procedure and post‐procedural measures have previously been described by our study group.^12^ Hundred milligram per day of acetylsalicylic acid (ASA) was prescribed indefinitely and 75 mg/day clopidogrel for at least 6 months after LAAC. A loading dose of 600 mg clopidogrel was administered, 250 mg, respectively, if clopidogrel had been taken before.

The study was carried out according to the principles of the declaration of Helsinki and was approved by the medical ethics committee II of the Faculty of Medicine Mannheim, University of Heidelberg, Germany. Informed consent was obtained from all patients.

### 2.2 Follow‐up cardiac computed tomography angiography imaging

cCTA imaging was performed 6 months after successful LAAC. A 2 × 192‐slice third generation dual‐source CT (Siemens Force, Siemens Healthineers, Forchheim, Germany) with a dual‐energy scan mode was used for all cCTA scans. Tube voltage 90 kV (tube A), 150 kV with tin filter (tube B) with topogram dependent tube current modulation for both tubes, detector collimation 2 × 192 × 0.6 mm and slice thickness 0.6 mm, increment 0.5 mm were acquisition parameters for the dual energy cCT. Retrospective ECG‐gating and bolus triggering technique with a region of interest (ROI) placed in the descending aorta and 100 HU threshold was performed in all cCTA acquisitions. About 80 cc of iodinated contrast material (Imeron 400, Bracco, Milan, Italy) were administered through an 18 G cubital catheter. Injection rate was 5 mL/second followed by a 50 mL saline flush. The systematic approach to evaluate implanted LAAC devices that has been recently described by the so called LOVE views, revealing optimal device‐related angulation allowing optimal evaluation of the device post implantation^13^ was applied.

### 2.3 Definitions

For measurement of the distances of the implanted occlusion device to NBS, the recently described LOVE axial and sagittal views^13^ were applied in all patients. Relevant NBS were defined: the MVA, the LPA, the LUPV and the Cx. LAA morphology was assessed for each patient and classified into one of four types suggested by Wang et al,^16^ namely windsock, chicken wing, cauliflower, and cactus.

### 2.4 Study endpoints

At cCTA visits, distances to neighboring structures were analyzed for each morphological type of LAA individually, as well as for both types of implanted LAAC devices (WATCHMAN and AMPLATZER AMULET). All patients were followed‐up regarding anticoagulant therapies and adverse clinical events for 12 months. This especially included arterial or venous thromboembolism, stroke or transient ischemic attack (TIA).

### 2.5 Statistical analysis and data availability

Statistical analyses were performed with IBM SPSS Statistics Version 21.0.0.0 (IBM, Armonk, New York). Metric variables are given as medians with inter quartile range (IQR). If n < 4, no IRQ is given. Numerical variables are given as total numbers with group‐related percentages. Distribution of metric variables among two or more groups was analyzed using Mann‐Whitney *U* test and Kruskal‐Wallis test by ranks, respectively. Distribution of nominal categories was analyzed with *χ* ^2^ test or Fisher exact test when more than 25% of cells in the contingency table had expected values of smaller than 5. A *P* value of <0.05 indicates statistical significance, a p value <0.10 indicates a statistical trend. The datasets generated during the present study are available from the corresponding author on reasonable request.

### 2.6 Ethical approval

All procedures performed in studies involving human participants were in accordance with the ethical standards of the institutional and/or national research committee and with the 1964 Helsinki declaration and its later amendments or comparable ethical standards. This study has been approved by the medical ethics committee II of the Faculty of Medicine Mannheim, University of Heidelberg, Germany.

## 3 RESULTS

### 3.1 Baseline characteristics

A total of 48 consecutive patients were included in the study and evaluated by cCTA using the LOVE view imaging algorithm 6 months (median 180 days, IQR 178‐180) after successful LAAC. Median age was 80 years, 25% were female. Median CHA_2_DS_2_VAsc score and median HAS‐BLED score were 4. Further baseline characteristics are shown in **[**Table 1 **]**.

**Table 1:** Baseline characteristics

Total number of patients	48
Female, n (%)	12 (25)
Age (years) median (IQR)	80 (75‐84)
Height (cm) median (IQR)	170 (167‐176)
Weight (kg) median (IQR)	80.0 (70.5‐90.0)
BMI (kg/cm^2^) median (IQR)	26.8 (24.6‐30.2)
Comorbidities, n (%)	
Hypertension	46 (96)
Diabetes mellitus	11 (23)
Prior stroke	8 (17)
Prior transient ischemic attack	2 (4)
Prior intracranial bleeding	5 (10)
Coronary artery disease	24 (50)
Peripheral vascular disease	3 (6)
Renal failure	16 (33)
Liver disease	4 (8)
AF type, n (%)	
Paroxysmal	23 (48)
Persistent	10 (21)
Permanent	15 (31)
Labile INR, n (%)	3 (6)
CHA_2_DS_2_VASc score^a^ median (IQR)	4 (3‐5)
HAS‐BLED score median (IQR)	4 (3–4)
Prior bleeding, n (%)	
Gastrointestinal	24 (50)
Intracerebral hemorrhage	6 (13)
Urinary	5 (10)
Others	6 (13)
Total	41 (85)

Abbreviations: AF, atrial fibrillation; BMI, body mass index; IQR, Inter quartile range.

a Following the 2016 guidelines of the European Society of Cardiology (ESC), sex category as independent risk factor was excluded from the original CHA_2_DS_2_VASc score.

### 3.2 Procedural characteristics

Median duration of LAAC device implantation was 90.5 minutes (IQR 70‐115). Contrast agent usage was 145 mL (IQR 110‐191) and median duration of fluoroscopy was 8.1 minutes (IQR 5.2‐12.8). Sedation was achieved by propofol injection alone in 5 patients (10%). Median dosage in those patients was 230 mg (IQR 210‐230). Forty‐three patients (90%) received additional midazolam injection. Median propofol dosage in those patients was 200 mg (IQR 160‐260) and median midazolam dosage was 5 mg (IQR 5‐7.5).

### 3.3 Distance to NBS

**Figure 1:**
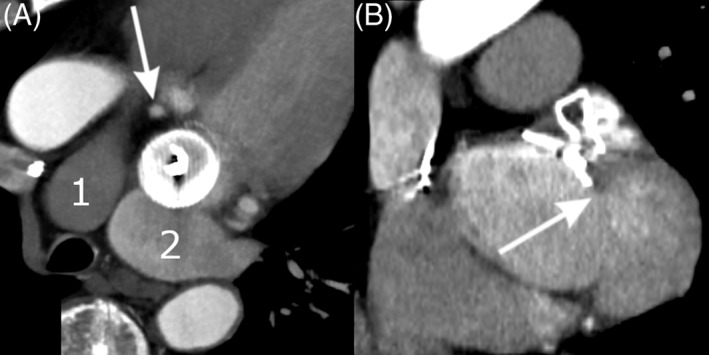
AMPLATZER AMULET device and left atrial appendage (LAA) neighboring structures, 6 months after implantation. A, LAA occluder view for post‐implantation evaluation (LOVE) axial view, showing pulmonary artery (1), left upper pulmonary vein (2), and circumflex artery (arrow). B, LOVE sagittal view, showing the relationship of the device to the mitral valve annulus

**Figure 2:**
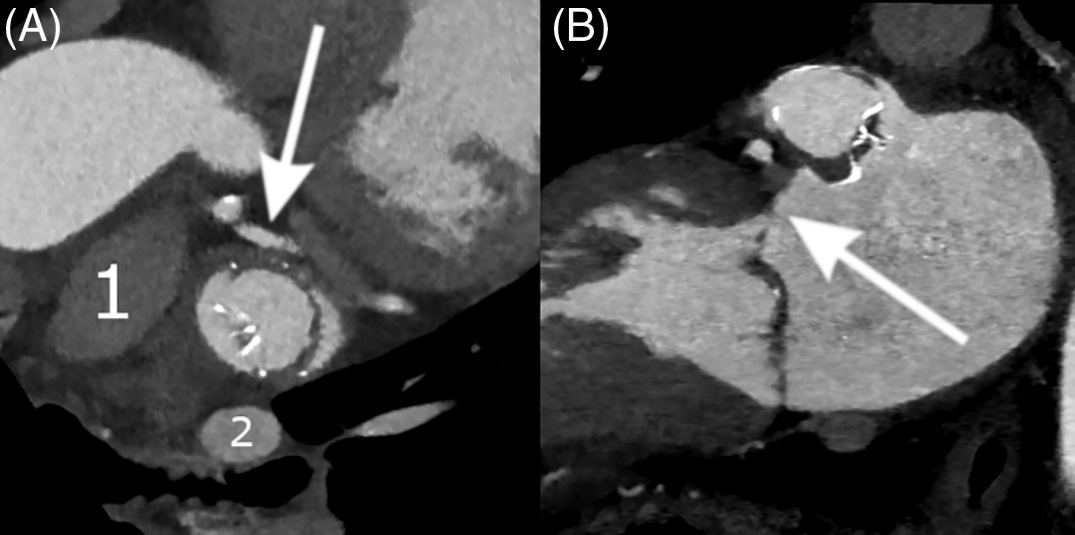
WATCHMAN device and left atrial appendage (LAA) neighboring structures, 6 months after implantation. Device is rotated in relation to the LAA orifice. A, LAA occluder view for post‐implantation evaluation (LOVE) axial view, showing pulmonary artery (1) left upper pulmonary vein (2), and circumflex artery (arrow). B, LOVE sagittal view, showing the relationship of the device to the mitral valve annulus

Distances to LPA, LUPV, and Cx were best visualized and measured in LOVE axial view, while the distance to MVA was demonstrated best in LOVE sagittal view **(**Figures 1 and 2). Overall median distances of implanted LAAC devices were 8.5 mm (IQR 6.3‐12.1) to MVA, 4.4 mm (IQR 2.3‐8.9) to LPA, 2.9 mm (IQR 2.0‐4.5) to LUPV and 2.8 mm (IQR 2.2‐3.6) to Cx.

### 3.4 LAA morphology and NBS

The prevalence of different LAA morphologies was 38% windsock, 33% chicken wing, 23% cauliflower, and 6% cactus type. Distances to MVA and LPA showed no relevant difference among the morphological types. In cauliflower type, devices were closest to the LUPV (1.7 mm) compared to windsock (2.6 mm), cactus (3.8 mm) and chicken wing (4.3 mm) types. A *P* value of 0.048 indicates statistical significance of those differences. A closer proximity to Cx was found in cauliflower (2.6 mm), chicken wing (2.7 mm), and cactus (2.7 mm) types compared to windsock (3.4 mm) type, but no statistical significance was reached (*P* = 0.439). Sizes of implanted devices and LAA landing zone were not significantly different between LAA morphologies. LAA morphologies had a significant difference in the type of implanted device. Windsock and cactus types had more AMPLATZER AMULET devices implanted, chicken wing and cauliflower had more implanted WATCHMAN devices, respectively **[**Table 2 **]**.

**Table 2:** LAA morphology related differences in distance to neighboring structures

Distance from (mm)	Windsock (n = 18; 38%)	Chicken wing (n = 16; 33%)	Cauliflower (n = 11; 23%)	Cactus (n = 3; 6%)	*P*‐value
Mitral valve annulus	7.5 (6.3‐12.5)	9.5 (7.2‐12.45)	8.3 (4.7‐1.3)	8.6	0.819
Pulmonary artery	4.1 (3.1‐8.9)	5.7 (2.1‐10.8)	3.7 (1.8‐7.5)	2.3	0.841
Left upper pulmonary vein	2.6 (2.0‐4.5)	4.3 (2.7‐6.0)	1.7 (1.0‐3.4)	3.8	**0.048**
Circumflex artery	3.4 (2.4‐4.4)	2.7 (2.2‐3.1)	2.6 (2.0‐3.2)	2.7	0.439
Device type (WM^*^/ACP^**^)	7 / 11	14 / 2	7 / 4	1 / 2	**0.014**
Device size	2.7 (2.4‐2.8)	2.4 (2.4‐2.7)	2.4 (2.4‐2.9)	2.2	0.307
Landing zone	18.0 (15.0‐22.3)	16.0 (14.5‐20.5)	16.0 (15.0‐18.0)	15.0	0.869

Values are presented as medians (IQR = interquartile range). If n < 4 no IQR is given. Bold type indicates statistical significance. ^*^WATCHMAN; ^**^AMPLATZER AMULET.

### 3.5 Devices and NBS

Twenty‐nine WATCHMAN devices and 19 AMPLATZER AMULET devices (60% vs 40%) were implanted. AMPLATZER AMULET devices showed closer proximity to the MVA (6.6 mm vs 10.0 mm, *P* = 0.001). AMPLATZER AMULET devices were also closer to LPA (3.9 mm vs 5.4 mm) and LUPV (2.5 mm vs 3.4 mm), although both differences were not statistically significant. In contrast, median distance of WATCHMAN devices to Cx was slightly smaller than in AMPLATZER AMULET devices (2.7 vs 3.1 mm, *P* = 0.323) **[**Table 3 **].**

**Table 3:** Device type related differences in distance to neighboring structures

Distance from (mm)	WATCHMAN(n = 29; 60%)	AMPLATZER AMULET(n = 19; 40%)	*P*‐value
Mitral valve annulus	10.9 (7.4‐14.0)	6.6 (4.9‐8.6)	**0.001**
Pulmonary artery	5.4 (2.3‐9.0)	3.9 (1.8‐9.1)	0.613
Left superior pulmonary vein	3.4 (1.6‐6.2)	2.5 (2.0‐3.8)	0.174
Circumflex artery	2.7 (2.2‐3.2)	3.1 (2.1‐4.3)	0.206

Values are presented as medians (IQR = interquartile range).

### 3.6 Adverse events

In the present study cohort, only one patient had a vascular adverse event during 12 months of follow‐up. The patient suffered from pulmonary embolism and subsequently had oral anticoagulation therapy reestablished 3 months after LAAC. This patient had a WATCHMAN device implanted and chicken wing type LAA morphology. Distance of the implanted device to the LPA was equal to the median distance of WATCHMAN devices (5.4 mm) and further away than AMPLATZER AMULET devices (3.9 mm); thus, direct correlation cannot be assumed. No stroke, transient ischemic attack (TIA) or other vascular adverse event occurred in this cohort.

## 4 DISCUSSION

The present observational study describes for the first time the distances of LAAC devices 6 months after implantation in relation to different LAA morphologies and device types, assessed by the standardized LOVE algorithm based on cCTA imaging in 48 consecutive patients.

It was demonstrated that LUPV and Cx are generally closest to LAAC devices and could thus be more prone to impairment. In correlation to device type, it was shown that AMPLATZER AMULET devices are closer to the MVA than WATCHMAN devices. This is accordant to existing literature, where MVA impairment was described as a possible complication only of AMPLATZER AMULET implantation.^17^ However, no serious adverse events that could be linked to impairment of NBS of the LAA were documented. All patients were treated by dual antiplatelet therapy for at least 6 months.

In the present study, varying distances of devices to NBS for the different LAA morphologies could be shown. However, the only statistically significant difference could be found in the LUPV, to which the closest median proximity of devices was shown in cauliflower type (1.7 mm). This supports previous findings, that LAA morphologies do not show significant differences in procedural success and adverse event rates.^18^, ^19^

The present study shows the possible uses and benefits of LOVE imaging in the assessment of LAA NBS in patients' follow‐up 6 months after LAAC. Distances between NBS and implanted device can be seen best using LOVE axial view. Higher resolution measurements of distances to and impairment of NBS can be performed by cCTA compared to TEE.

There is only little knowledge on the consequences of LAAC device implantation for NBS. There are, however, case reports of relevant compression of Cx, leading to ST‐elevations,^8^, ^11^ LPA perforation,^9^, ^10^ and pulmonary vein compression.^8^, ^11^ Direct contact to or beyond the MVA could lead to valvular dysfunction. In this cohort, however, no adverse events originating from impairment of NBS were found. Further assessment of clinical consequences of NBS impairment is still needed. Standardized LOVE cCTA imaging could facilitate comparability of studies assessing this topic. Differences of LAA morphologies and implanted devices in distances to NBS should be accounted for.

Other uses of cCTA have been proposed in the planning of LAAC and assessment of early and mid‐term outcomes of device implantation,^6^ three‐dimensional geometric CT analysis of the LAA could prove to be effective for prediction of PDL.^20^ Aspects in favor of the LOVE cCTA protocol are non‐invasiveness and higher resolution measurements of CT compared to TEE evaluation of LAAC, paired with higher objectivity and reliability of a standardized protocol.

### 4.1 Limitations of this study

This is a prospective observational study of 48 consecutive patients with successful LAAC undergoing standardized cCTA imaging protocol at mid‐term follow‐up. Because of the small sample size, all results of this study can only be of hypothesis generating character. Further investigations with larger sample sizes are needed to draw any definitive conclusions.

## 5 CONCLUSION

The distance of LAAC devices to neighboring structures of the LAA is dependent on LAA morphology and type of implanted device. Left upper pulmonary vein and circumflex artery are generally closest to LAAC devices and might thus be more prone to impairment. AMPLATZER AMULET devices are closer to the mitral valve annulus than WATCHMAN devices. Further investigation on clinical consequences of close proximity of LAAC devices to NBS is needed. A standardized cCTA imaging protocol is an eligible tool to accurately measure distances to LAA NBS.

## CONFLICTS OF INTEREST

The authors declare no potential conflict of interests.
